# Core microbial populations drive longitudinal development of the hindgut bacterial microbiota in Jersey calves

**DOI:** 10.1128/spectrum.01878-26

**Published:** 2026-10-09

**Authors:** A. Post, A. Yadlapalli, T. Webb, A. Zhang, A. Yelampalli, K. Challa, N. Indugu, D. W. Pitta

**Affiliations:** 1 Department of Clinical Studies, New Bolton Center, University of Pennsylvania School of Veterinary Medicine 70736, Kennett Square, Pennsylvania, USA; Foshan University, Foshan, China

**Keywords:** dairy calf microbiome, jersey cattle, longitudinal microbiome development, microbial succession, microbial–metabolic networks, acetate metabolism

## Abstract

The early-life gut microbiome represents a critical window during which microbial succession may exert lasting effects on host metabolism and productivity. While gut microbiome–host phenotypic associations have been extensively studied in Holstein calves, such relationships are rarely reported in alternative breeds, including Jersey calves. We hypothesized that Jersey calves would exhibit age-dependent community shifts characterized by core microbial populations and breed-associated bacterial networks that may contribute to metabolic phenotype. Fecal samples were collected from dams post-calving, and Jersey calves (*n* = 12) were sampled longitudinally at 24–48 h and weekly through 12 weeks. Bacterial communities were profiled using the V1–V2 region of the 16S rRNA gene. The number of observed ASVs and Shannon diversity increased with age (*P* < 0.001), and Beta diversity revealed age-dependent structuring (weighted *R*² =0.12, *P* = 0.023; unweighted *R*² =0.37, *P* = 0.001), with calf communities gradually converging toward dams. Taxonomic analyses revealed a conserved core microbiome comprising 34 taxa accounting for over 90% of total community abundance. Neonatal samples were dominated by facultative anaerobes, including *Escherichia* and *Streptococcus*, with early colonization by *Blautia* and *Collinsella*. Calves then exhibited transient enrichment of milk-glycan-associated taxa, including *Lactobacillus* and *Bacteroides*. Weeks 3–7 stages saw preeminence of acetogens (*Blautia*, *Dorea, Collinsella*), followed by later enrichment of adult-associated fiber-degrading taxa. Collectively, these findings demonstrate a consistent age-dependent pattern of hindgut microbiome maturation among Jersey calves. The taxonomic patterns provide a foundation for investigating potential links between early-life microbial ecology and breed-specific metabolic phenotypes.

**IMPORTANCE:**

The neonatal period represents a critical window for microbial community assembly and interaction with the host. While microbial succession in Holstein calves has been studied extensively, little is known about microbiome assembly in Jersey calves. Herein, we performed longitudinal profiling of the hindgut microbiome of Jersey calves from birth through twelve weeks of age. Microbial communities underwent a structured succession broadly resembling patterns reported previously in Holsteins, with network analyses revealing microbial assemblages that shift with diet throughout the pre- and postweaning periods. Jersey calves also exhibited a notably elevated abundance of several acetate-associated taxa during early life. These findings provide the first detailed characterization of hindgut microbiome assembly in Jersey calves and identify microbial community structures potentially linked to breed-specific metabolic phenotypes. Such information is critical for designing microbiome-targeted strategies to improve calf growth, health, and feed efficiency, and for influencing milk components.

## INTRODUCTION

The microbiome plays a critical role in the health of all mammals, with particular significance in cattle, where rumen microbes facilitate the breakdown of complex feedstuff into nutrients essential for growth and milk production. This microbial ecosystem is heavily influenced during early life by factors, such as maternal influences, colostrum intake, housing, feed type, environmental conditions, and seasonal variation (1).

In dairy calves, the composition of the early-life microbiome has been extensively studied, particularly in the rumen and hindgut (2–7). These studies reveal that microbiomes undergo dynamic and significant shifts during the preweaning period. However, most investigations have focused on Holstein calves raised under confinement systems, reflecting the dominant breed and rearing strategy for U.S. dairy production ((8)). Given the heritability of microbial traits and the influence of diet and management, it is reasonable to expect that different breeds and production environments foster distinct microbial communities. Jerseys are increasingly used in pasture-based and hybrid grazing systems due to their smaller body size, superior feed efficiency, and high milk solids content (8, 9). Jersey milk, rich in fat and protein, supports premium dairy products such as butter and ice cream, with both host genetics and microbial metabolism influencing this phenotype (10). Only two studies to date have examined Jersey calf microbiomes (11, 12), both limited to the first 3 weeks of life and focused on health status differences. Thus, little is known about the temporal development of the Jersey calf microbiome throughout the full preweaning period.

Early-life microbial colonization likely reflects the admixture of unique genetic traits and environmental conditions of calf environments. In this fluid phase of ecology, differences in nutritional composition and environmental exposure are met by genetic selection pressures on the microbiome shaping long-term microbial community composition, driving differences in forage utilization, metabolite production, and milk yield and composition (13, 14). To date, no studies have comprehensively characterized temporal changes in the fecal microbiome of Jersey calves throughout the pre- and postweaning periods. Previous work from our group demonstrated that the Holstein calf microbiome pre-weaning is variable but stabilizes throughout early life, with increasing abundance of fiber and starch degraders, becoming an adult-like microbial community at weaning (3). Further (15) established that bacterial-archaeal and bacterial-bacterial syntrophies originate early during the preweaning period, and these relationships orchestrate overall community architecture. Recent work confirmed that in adulthood, Jersey and Holstein cows differ in taxonomy and microbial function, with Jersey cows generating more hydrogen (H_2_), leading to increased production of acetate and methane compared to Holsteins (16). Acetate, in turn, has been recognized as microbial as a primary precursor for lipogenesis, including in the mammary gland.

When assessing both the microbial and phenotypic differences between Holstein and Jersey cows, with our understanding of the centrality of the early-life period, it is tempting to speculate that the unique microbial community of the Jersey breed and its resultant fermentation establish in the neonatal phase, and this in turn contributes a unique role in sustained breed differences, including fat metabolism. Based on this, we hypothesized that the Jersey calf microbiome undergoes structured, longitudinal shifts during early life, characterized by the emergence of core microbial populations, and that breed-specific taxonomic configurations may reflect distinct microbial metabolic potential, consistent with known breed-associated physiological traits. This study is the first to describe age-related fluctuations in the fecal microbiome of Jersey calves grown on pasture from birth through 12 weeks of age. The findings presented here will lay the groundwork for understanding Jersey calf microbiome dynamics, providing potential microbial targets for improving health, nutrient utilization, and understanding breed-specific traits in dairy production.

## MATERIALS AND METHODS

### Study design and animal husbandry

Jersey calves (*n* = 12) were enrolled at birth from a private farm in Maryland between February and April 2025. Calves received hand-fed colostrum, consisting of 2 quarts administered twice during the first 24 h, the first within 6 h of birth, using primarily frozen colostrum. Calves weighed an average of ~65 lb at birth (range: 55–80 lb) and remained with the dam for approximately 12 h before entering the standard farm rearing program. Thereafter, calves were fed a combination of whole raw milk and milk replacer and were housed individually in outdoor calf hutches until approximately 14 days of age. Following this period, calves were transitioned to group housing with age-matched peers. All calves had *ad libitum* access to water throughout the study. Calf starter was introduced at 7–10 days of age, although minimal intake was observed until approximately 3 weeks of age, and free-choice hay was provided beginning at 4 weeks of age. Calves were weaned by 60 days of age, with an estimated average body weight of 140 lb at weaning. All procedures were approved by the University of Pennsylvania Institutional Animal Care and Use Committee (protocol #: POAP 725).

### Extraction of genomic DNA and 16S rRNA sequencing

Fecal samples were obtained via rectal stimulation and stored immediately at −20°C until all samples were collected. Samples were then placed on ice for transport to the laboratory, at the New Bolton Center, University of Pennsylvania, where they were again stored at −20°C until extraction for DNA (approximately 1.5–2 months after collection

Approximately 250 mg of feces from all samples were processed to extract genomic DNA using the repeated bead beating and column (RBB + C) method with added purification steps using the QIAamp Fast DNA Stool Mini Kit (17). For samples containing meconium (day 2 and week 1), only 50 mg of feces was used to ensure a high-quality DNA product. The extracted DNA had the V1–V2 region of the bacterial 16S rRNA gene PCR-amplified in triplicate, were multiplexed, and PCR amplified as described previously ([3, 4]). Amplicons from each DNA sample were combined and quantified using a Quant-IT Broad Range assay kit (kit), and then libraries were pooled in an equimolar concentration, and purified using Agencourt AMPure XP Beads (Beckman Coulter; Brea, CA, USA). Sequencing was then performed using an Illumina MiSeq platform at the PennCHOP Microbiome Core in Philadelphia, Pennsylvania.

### Bioinformatic analysis

The bacterial 16S rRNA sequencing data were analyzed using the QIIME2 pipeline (version 2020.6 (18);), as described in Hennessy et al. (3). Briefly, the sequences were separated by sample (demultiplexed) and quality filtered. Amplicon sequence variants (ASVs) were identified using the DADA2 plugin. A phylogenetic tree was built using MAFFT (19) for sequence alignment and FastTree 2 (20) for tree construction. The ASVs were classified by comparing them to the V1–V2-specific Greengenes reference database (v13.8) using a naive Bayes classifier.

Alpha diversity was calculated to measure diversity within each sample. The number of observed ASVs indicated the number of different taxa present (richness). The Shannon diversity index combined the number of taxa and their relative abundances to provide an overall estimate of diversity. Beta diversity was calculated to compare differences between samples. Weighted UniFrac accounted for the abundance of taxa, while unweighted UniFrac considered only their presence or absence. Before performing diversity analyses, the data were normalized to 8,644 reads per sample using the “qiime diversity” function.

### Statistical analysis

All statistical analyses were performed in R (version 4.4.3). The measured alpha diversity matrices, including observed ASVs and Shannon diversity, were compared between timepoints using the Kruskal-Wallis test or Wilcoxon Rank Sum test, as appropriate. Permutational multivariate analysis of variance (PERMANOVA) was used to test for differences in beta diversity among timepoints based on weighted and unweighted UniFrac distance matrices. PERMANOVA models were restricted to calf samples with AnimalID specified as a strata variable to account for repeated measures. Dams were included in ordination plots to visualize convergence of calf bacterial communities toward adult-like communities. Pairwise PERMANOVA analysis (vegan::adonis2) was conducted to compare each calf age group against dams.

To identify differentially abundant genera, we employed a consensus approach incorporating multiple statistical frameworks, including linear mixed-effects modeling (lme4, lmer), analysis of composition of microbiomes with bias correction 2 (ANCOM-BC2 [21]), and microbiome multivariable associations with linear models (MaAsLin3 [22]). For the linear mixed-effects models, genus-level relative abundances were adjusted by adding a pseudocount of 1 and log2-transformed prior to analysis. For calf-only analyses, timepoint was included as a fixed effect and AnimalID was included as a random effect to account for repeated measures. For models including both calves and dams, group (calf or dam) was included as a fixed effect with the same random effect structure. Genera were considered differentially abundant if they were identified as significant by at least two of the three methods.

To identify the “core genera,” we exclusively used genus-level count tables from calf samples to construct a phyloseq object (phyloseq v1.55.1 [23]), separating out rare taxa with a 0.5% relative abundance filter. To classify our “core taxa,” remaining samples were merged by Animal ID and filtered for taxa present in ≥80% of calves. The contribution of core taxa to community structure was quantified by calculating their combined relative abundance per sample and through comparison of their contribution to microbial ecology by performing PERMANOVA (adonis2, vegan package) tests on Bray–Curtis dissimilarities with a model of the form: distance ~ age. To assess whether core taxa explained more community structure than non-core taxa, we performed 1000 iterations of random sets of non-core genera of equal size to the core set without replacement, and recomputed Bray–Curtis distances and PERMANOVA models. We took the mean of the R² values from all iterations and compared them to the core R².

To define temporal clustering of the “core taxa,” we rarefied calf fecal samples to the lowest sample sequencing depth using phyloseq, trimming low-abundance genera after rarefaction. Rarefied samples were then merged by time point, and relative abundance distances between taxa were calculated using Euclidean distance, performing hierarchical clustering with Ward’s minimum variance method. To determine the optimal number of temporal clusters, we employed the gap statistic (R cluster package) using a *k*-means clustering algorithm. The optimal number of clusters (*K*) was chosen using the Tibshirani 1-SE rule (24), selecting the smallest *K* for which the observed gap value was within one standard error of the maximum. Heatmaps of genus relative abundance across timepoints were generated using the ComplexHeatmap package in R.

Microbial association networks were inferred for the core taxa using the unrarefied core genus count and the SPIEC-EASI function (SpiecEasi package in Kurtz et al. [25], employing the Meinshausen–Bühlmann neighborhood selection method. The final adjacency matrix was extracted and converted to an igraph object, and clusters of tightly connected genera were identified using a community detection algorithm with igraph. Network visualizations were generated using ggplot2, with nodes colored by taxonomic order, and major network modules highlighted.

Spearman correlation analysis was performed on genera that differed significantly between calves and dams and exhibited significant age-related changes. Genera were considered present if their average percentage across all time points was at least 0.01%, and correlations were considered significant if *q* ≤ 0.05. For all data, differences were considered significant at *P* and *q* values ≤ 0.05 and trends at 0.05 < *P* or *q* ≤ 0.10. Adjusted *P* values are indicated using a *q*.

## RESULTS

### Changes in alpha diversity (within-sample variation) metrics with age

**Fig 1:**
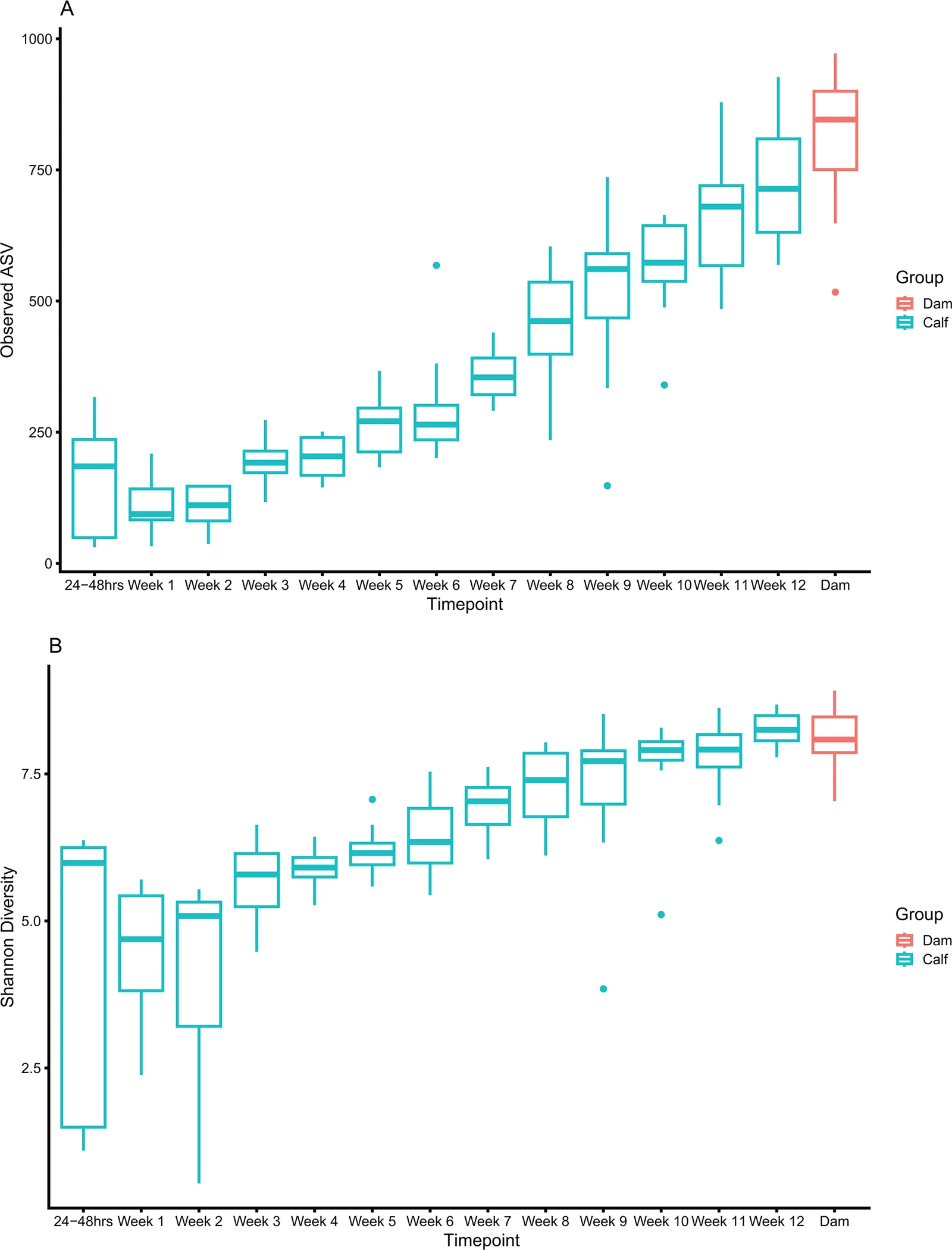
Alpha diversity or within-sample variation across the study period. Individual results from 12 Jersey calves and dams were grouped by age for analysis of (**A**) the number of amplicon sequence variants (ASV), and (**B**) the distribution of ASVs within each community (Shannon diversity index). Boxes represent the interquartile range (IQR) between the first and third quartiles, with a central horizontal line inside the box defining the median, and whiskers defining 1.5x IQR in either direction. All calves were weaned between weeks 8 and 9 (60 days).

Alpha diversity increased significantly with age by Kruskal-Wallis test, both for observed ASVs (*P* < 0.001; Fig. 1A) and Shannon diversity (*P* < 0.0001; Fig. 1B). Wilcoxon rank sum comparisons demonstrated that the significant divergence in richness is mostly present between earlier timepoints (24–48 h to week 3) to week 7 and beyond, with similar results for Shannon Diversity, with both metrics increasing with age such that week’s 7 through 9 were significantly lower than week 12, which was not significantly different from post-calving dam samples. Both metrics also demonstrated that calves had significantly lower richness and Shannon diversity than dams (*P* < 0.001).

### Comparison of fecal bacterial communities (beta diversity) by age and phenotypic factors

**Fig 2:**
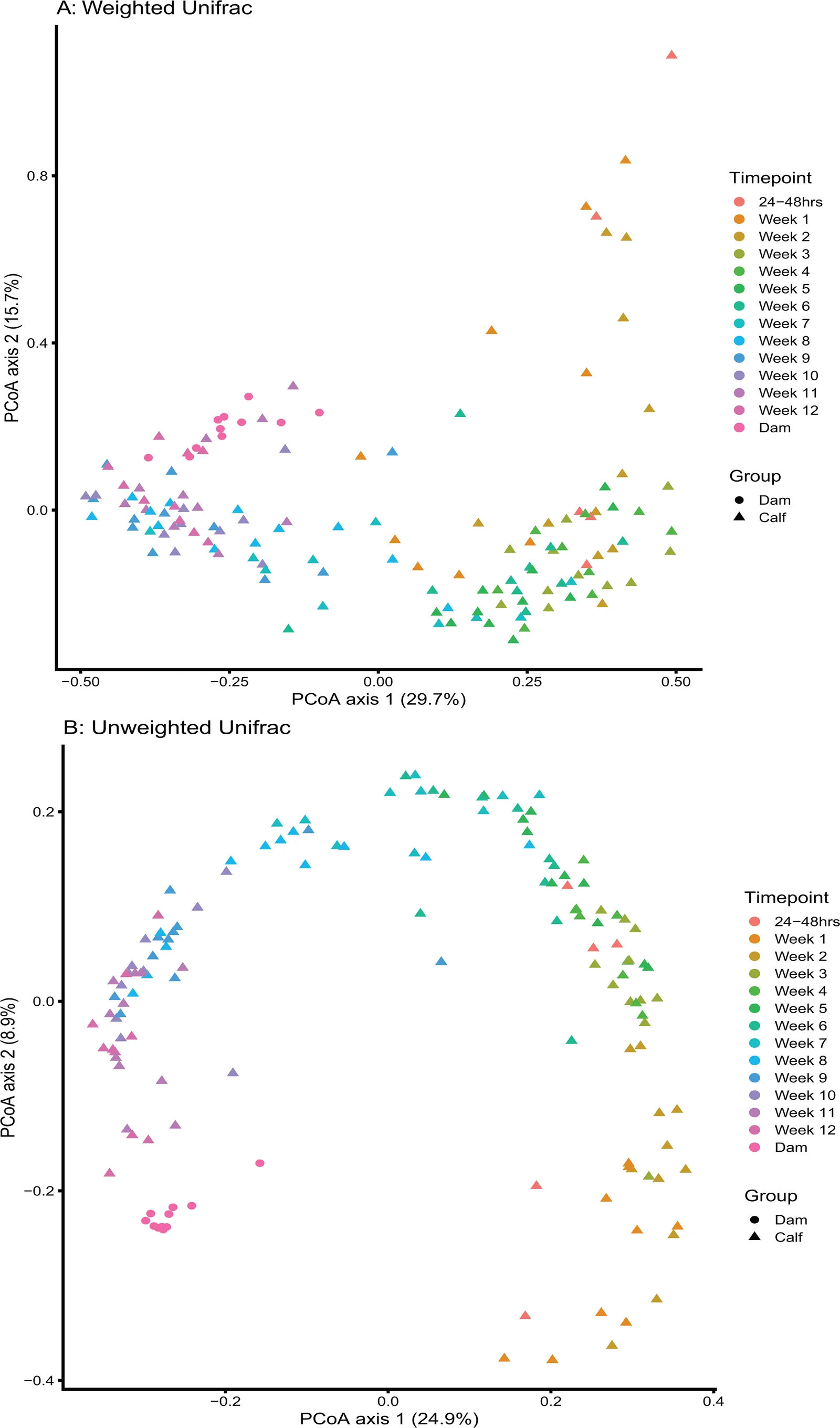
Beta diversity or between-bacterial community comparison across age in Jersey calves and dams. Principal coordinate analysis (PCoA) of (**A**) weighted and (**B**) unweighted UniFrac distances. Differences were assessed for significance by PERMANOVA. All calves were weaned between weeks 8 and 9 (60 days).

Both weighted and unweighted UniFrac analyses revealed significant shifts in microbial community composition as calves aged (weighted UniFrac: *R*² = 0.12, *P* = 0.023; unweighted UniFrac: *R*² = 0.37, *P* = 0.001; Fig. 2). Calf fecal communities were distinct from those of dams throughout the study. Immediately after birth, calf and dam communities diverged progressively through week 5 (24–48-week 1, *R*² = 0.36–0.38; *P* = 0.001, weeks 1 through 5 *R*^2^ = 0.046–0.56). Thereafter, calf communities began converging toward the adult-type microbiome, showing increasing similarity to dams by 12 weeks of age (weighted: *R*² =0.36, *P* = 0.001, unweighted: R² = 0.21, *P* = 0.002).

Calves remained significantly more dispersed than dams across the preweaning period, consistent with higher inter-individual variability during early colonization. Dispersion peaked around week five for both weighted (*R*² = 0.24) and unweighted (*R*² = 0.22) UniFrac distances, followed by convergence and reduced variability by weeks 11–12. Betadisper and Tukey post hoc analyses indicated that this early dispersion was driven primarily by 24–48 h to week 1 contrasts with timepoints of week three and beyond, suggesting rapid microbial differentiation during the initial colonization phase. Unweighted UniFrac distances revealed significant dispersion between calves and dams, but not between time points, suggesting compositional shifts rather than purely abundance-based changes (Fig. 2).

### Taxonomic composition of the calf microbiome

*Firmicutes, Bacteroidetes*, and *Actinobacteria* were dominant, together maintaining approximately 97% or greater of relative abundance between weeks 2 and 8 (Fig. S1). Very early life was impacted but a significant enrichment in *Proteobacteria* with a relative abundance of 21.4% at 24–48 h, falling to 5.9% at week 1 and remaining lower, ~0.5% abundance, for the remainder of the study. *Bacteroidetes* experienced a transient spike between 24 andd 48 h and week 1, then fell dramatically, again beginning to climb to about 12% abundance at weeks 7 through 12. In contrast, *Actinobacteria* experienced a notable decline in abundance at week 1, which then recovered and peaked at approximately 26% by week 5, decreasing thereafter throughout week 12 (Fig. S1). Coinciding with the peak in *Actinobacteria*, animals began to enrich in *Tenericutes*, which would continue throughout week 12, peaking at approximately 3%. *Cyanobacteria* exhibited increases in the later timepoints, particularly weeks 11 and 12, wherein it spiked four- to fivefold relative to the several preceding weeks. In comparison to calves, dams were significantly enriched in several phyla and demonstrated enrichment in *Firmicutes*, having a relative abundance of 90%, *Spirochaetes,* and *TM7* independent of calf age (*q* < 0.001).

At the genus level, calves were broadly distinct from dams, displaying enrichment in several key taxa (Fig. S2). Linear mixed-effects modeling identified significant enrichment of *Blautia*, *Dorea*, *Eubacterium*, *Collinsella*, *Bacteroides*, *Sharpea*, *Streptococcus*, *Parabacteroides*, *Eubacterium*, *Lactobacillus*, *Lactococcus*, *Ruminococcus*, *Faecalibacterium*, *Prevotella*, *Subdoligranulum*, *Odoribacter*, and *Clostridium* in calves relative to dams (LMER, *P* < 0.05). Multivariate association testing (MaAsLin3) confirmed significant abundance differences for *Odoribacter*, *Eubacterium*, *Bacteroides*, *Pseudoramibacter/Eubacterium*, *Sharpea*, *Streptococcus*, and *Lactobacillus* (*P* ≤ 0.05 or trending at *P* ≤ 0.10). ANCOM-BC2 further supported differential abundance of *Eubacterium*, *Lactobacillus*, *Bacteroides*, and *Parabacteroides* between calves and dams. Conversely, across all methods, dams were enriched in *02d06, Turicibacter, Butyrivibrio,* and unclassified members of the families *Peptostreptococcaceae* and *Christensenellaceae*.

**Fig 3:**
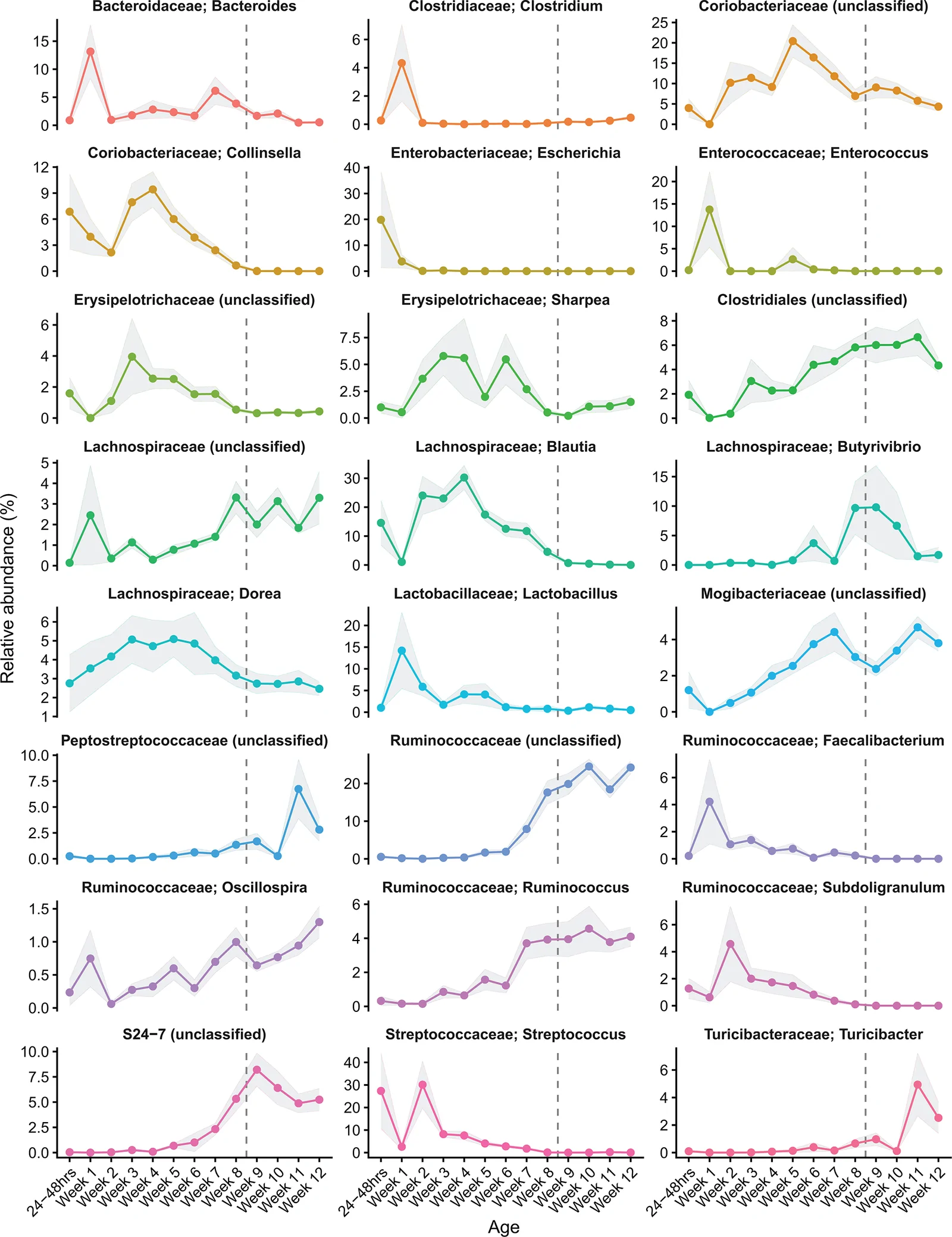
Temporal abundance of a subset of abundant core bacterial lineages in calves. Individual results of abundance of core microbial species (prevalence > 80%, mean abundance > 0.5%) from the 12 calves were grouped by age and plotted across the study period. Dots and lines track mean abundance, and ribbons represent standard error of the mean (SEM). Taxonomic features include family name and genus-level assignments and unresolved lineages reported at the highest assigned family or order. The vertical dashed line indicates the age by which all calves had been weaned (60 days). Relative abundances of all detected taxa are provided in Table S1.

Community dynamics were evidently structured by age effects, with significant changes in both the selected abundant and/or age-associated core taxa displayed in Fig. 3 and additional taxa reported in Table S1. The earliest timepoint, 24–48 h, was dominated by two facultative anaerobes, *Escherichia* and *Streptococcus*, making up close to 50% abundance, followed by several other commensals, such as *Blautia* (14.6%)*, Collinsella* (6.9%)*, Dorea* (2.75%)*, Clostridium* and *Sharpea* (both between 1% and 2%), and unclassified members of the families *Coriobacteriaceae* (4%)*, Lachnospiraceae, Mogibacteriaceae, Erysipelotrichaceae*, and order *Clostridiales* (all between 1% and 2%) (Fig. 3). As calves progressed to 1 week of age, the microbial community demonstrated profound changes, including drops in unclassified taxa of *Coriobacteriaceae*, *Clostridiales*, *Mogibacteriaceae,* and *Erysipelotrichaceae* (*q* < 0.05, all statistical methods; Table S1), and the genera *Streptococcus* (2.58%), *Blautia*, *Collinsella* (3.96%) (*q* < 0.05 lmer), and *Escherichia* (3.73%) to below 1% (Fig. 3). This was accompanied by enrichment in *Lactobacillus* (14.2%), *Enterococcus* (13.8%), *Bacteroides* (13.2%, *q* < 0.05 lmer and *q* = 0.077 ANCOMBC2), unclassified *Lachnospiraceae* (6.64%), *Butyricoccus* (*q* < 0.05 multiple methods), *Clostridium* (*q* < 0.05 lmer), and *Faecalibacterium* (*q* < 0.05 lmer), all ranging between 4.2% to 4.7%. Other than continued drops in *Collinsella*, week 2 saw a reversion to a pattern of abundance similar to 24–48 h, with drops in several taxa from *Clostridiales*, including *Faecalibacterium*, *Mogibacteriaceae, Butyricoccus (q <* 0.05 lmer)*, Lachnospiraceae* (*q* < 0.05 lmer), and unclassified *Clostridiales*, all falling to or below 1% abundance. *Lactobacillus* (5.87%) and *Bacteroides* (~1%, *q* < 0.05 lmer) fell in abundance as well. The major abundant taxa during this time included *Streptococcus* (30.1%), *Blautia* (24%, *q* < 0.05 lmer)*, Coriobacteriaceae* (10.2%, *q* < 0.05 lmer)*, Subdoligranulum* (*q* < 0.05 lmer), *Ruminococcus* (*q* < 0.05 Lmer and MaAsLin3), *Dorea* (*q* < 0.05 Lmer and MaAsLin3), *Sharpea* (*q* < 0.05 lmer), *Collinsella*, and *Erysipelotrichaceae* (Fig. 3). Weeks 3 through 7 were marked by the persistent dominance of the taxa *Blautia*, *Collinsella*, *Sharpea*, *Dorea*, *Streptococcus*, *Bacteroides* (Fig. 3), and several unclassified taxa from the families Coriobacteriaceae, Lachnospiraceae, and the order *Clostridiales*.

The end of this period, moving into week 8 and 9, saw more large changes in community dynamics, as significant decreases (by ANCOMBC2 and MaAsLin3) in the abundance of *Blautia*, *Collinsella*, *Streptococcus*, *Subdoligranulum*, *Lactococcus*, and *Clostridium* from the *Erysipelotrichaceae* family, along with numeric decreases in *Dorea* and *Bacteroides,* all of which would continue throughout the remainder of the study period (Fig. 3). Several other taxa began to enrich relative abundances during week 8 and 9, including *Ruminococcus*, *Buryrivibrio*, and unclassified taxa from the families *Lachnospiraceae*, *Ruminococcaceae*, *S24-7*, and *Rikenellaceae* (*q* < 0.05 MaAsLin3 and/or ANCOMBC2, except for *Butyrivibrio;* Fig. 3). Weeks 10 and 11 saw the continued prominence of *Ruminococcus*, *Butyrivibrio*, and unclassified members of *Ruminococcaceae*, *Coriobacteriaceae*¸ and *Lachnospiraceae*. These later timepoints were also marked by the enrichment of unclassified *Peptostreptococcaceae* (6.74%) and the genus *Turicibacter* (4.94%). By week 12, the microbial community was heavily enriched in unclassified taxa from the order *Clostridiales* (18.9%) and families *Ruminococcaceae* (27.7%), *S24-7* (5.2%), *Coriobacteriaceae* (4.3%), *Mogibacteriaceae* (3.8%), the genera *Ruminococcus* (4.1%), with unclassified *Lachnospiraceae, Peptostreptococcaceae,* and the genera *Turicibacter, Dorea, and Butyrivibrio* all between 1.7% and 3.8% abundance (Fig. 3; Table S1 for *Clostridiales and Mogibacteriaceae*).

The recurring pattern across animals suggested to us that there is a conserved microbial succession pattern among the cohort of Jersey calves studied, thus, we aimed to define a set of microbes that may direct the succession of the early-life microbiome. We defined this core microbiome as recurring taxa present in at least 80% of our sample cohort, with an average relative abundance of 0.5%. 34 core taxa were defined, representing 90.8% of the total relative abundance in all animals, and when we examined the contribution of these microbes to successional patterns, they explained substantially more community variance through all sampling timepoints (PERMANOVA *R*² = 0.444; *P* < 0.001) than 1,000 permutations of randomly selected non-core genera (mean *R*² = 0.291). Looking at the succession of these core microbes through the preweaning to postweaned period, we identified six primary temporal clusters (Fig. S2): (i) 24–48 h, (ii) week 1, (iii) week 2, (iv) weeks 3 and 4, (v) weeks 5 through 7, and (vi) week 8 onwards to 12 weeks of age; matching the consistent grouping patterns of microbes as described above.

**Fig 4:**
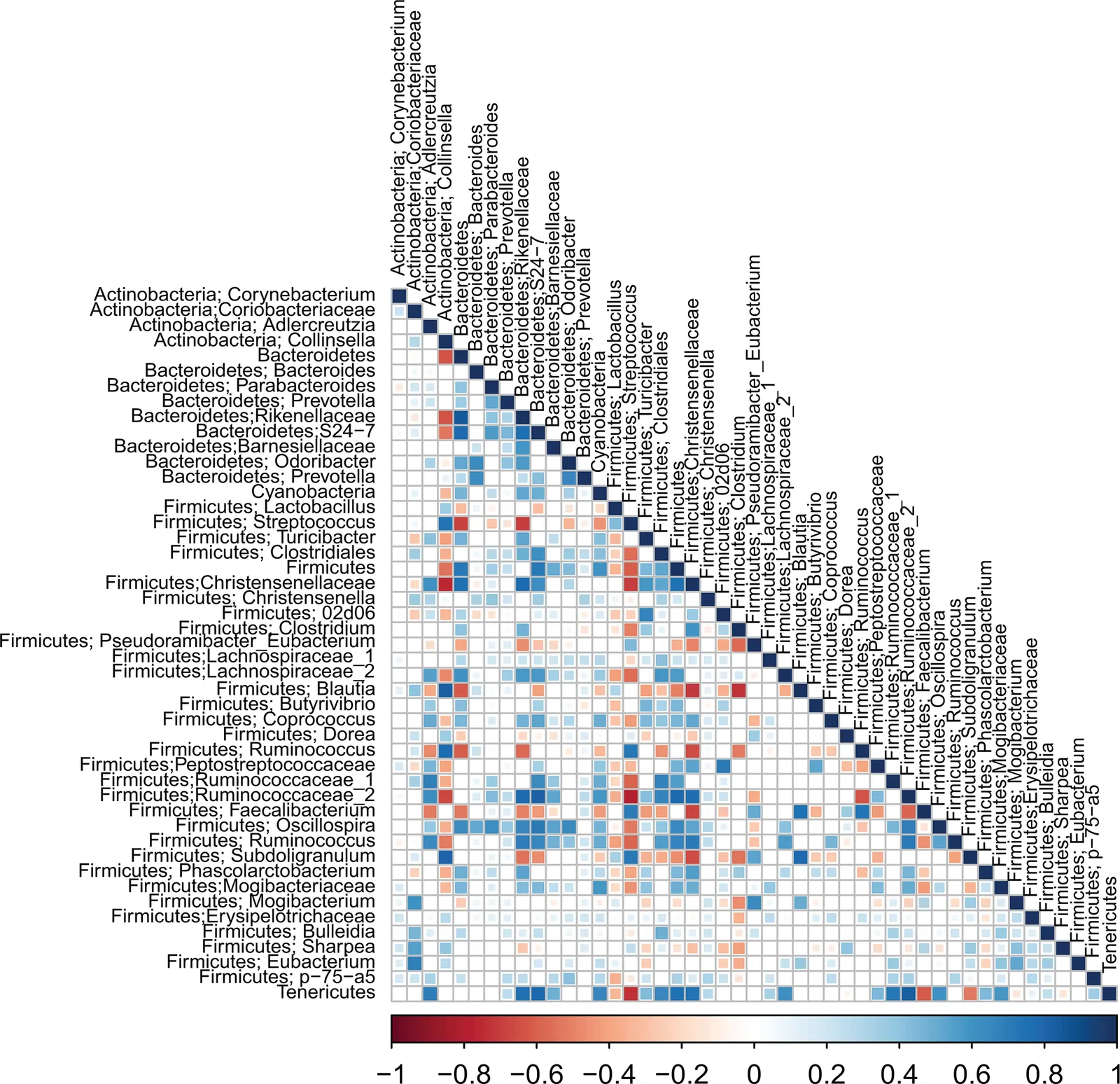
Associations among bacterial taxa as revealed by Spearman’s correlation plot. Only statistically significant correlations (adjusted *P* < 0.05) are shown. Positive and negative associations are indicated in navy blue and red, respectively. Taxa are indicated as phylum name followed by lowest available classification level.

To build upon the associations between age and a changing core bacterial group, a Spearman correlation analysis was performed, focusing on bacteria that differed significantly between calves and dams and exhibited significant age-related changes (Fig. 4). Broadly, two prominent groupings of correlation were observed. The first group consisted of strong positive correlations (Rho > 0.7) between *Blautia, Collinsella, Lachnospiraceae, Ruminococcus*, *Faecalibacterium,* and *Subdoligranulum*. This group often demonstrated moderate to strong (0.4 < Rho < .7) negative correlations to the second grouping of *Ruminococcaceae*, *Christensenellaceae*, *Rikenellaceae*, *Bacteroidales*, *Clostridiaceae*, *Clostridium*, *Clostridiales*, and *S24-7,* which in and of themselves were often strongly positively correlated to one another. *Oscillospira* showed a strong positive correlation with S24-7 and *Ruminococcaceae*, and a moderate negative correlation with *Streptococcus. Bacteroides* was also positively correlated with *Oscillospira, Prevotella,* and *Odoribacter*. Of the genera that were enriched late in calf development, *Turicibacter* had moderate positive correlations with 02d06, Christensenellaceae, and Butyrivibrio, and moderately negative correlations with *Faecalibacterium*, *Blautia*, and *Collinsella. Peptostreptococcaceae* similarly demonstrated a strong positive correlation with 02d06, as well as moderate positive and negative correlations with *Butyrivibrio* and *Christensenellaceae*, and *Faecalibacterium* and *Collinsella*, respectively.

**Fig 5:**
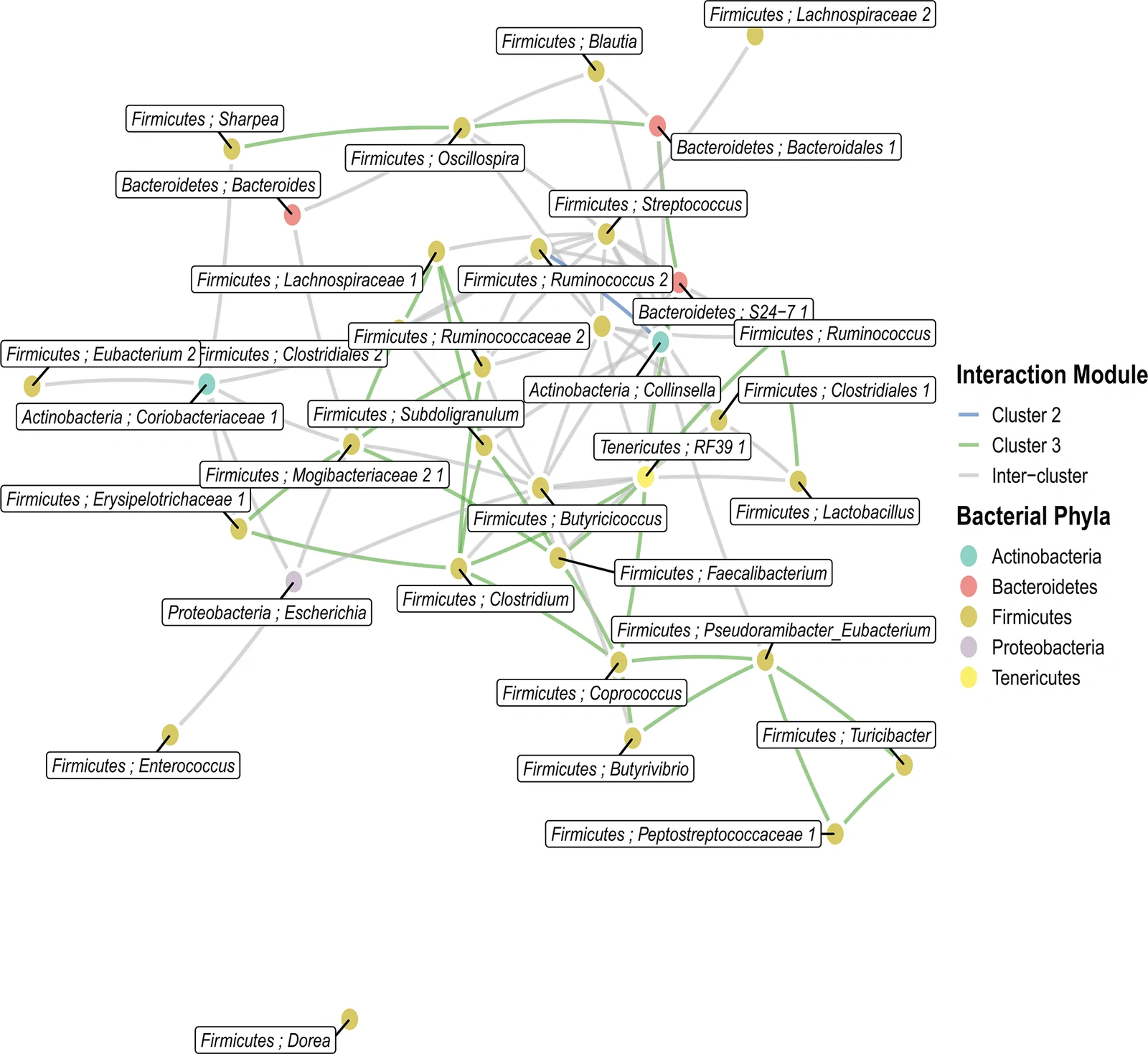
SpiecEasi-inferred ecological network of Jersey calf core taxa. Core taxa, those with a prevalence > 80% and mean relative abundance > 0.5%, were used to construct a sparse inverse covariance network. Nodes represent taxa (colored by bacterial order); edges represent inferred associations. Cluster structure (Ward’s D2; *k* determined by gap statistic) is indicated by colored intra-cluster edges; inter-cluster edges are shown in gray.

With several significant correlations between two predominant groupings of genera, we sought to determine whether the correlations we observed match networks formed by core members of the early-life microbiome which are displayed as nodes. Using SpiecEasi we generated co-occurrence networks defined by eight modules, composed largely of one major and one minor module (Fig. 5). Cluster 3, the largest, consisted of 21 of the 34 core taxa, including: *Blautia*, *Turicibacter*, *Butyrivibrio, Bacteroides RF39*, *Corynebacterium, Escherichia*, *Dorea*, *Oscillospira*, unclassified taxa from the families *Rikenellaceae*, and *Ruminococcaceae*, and from the orders *Bacteroidales* and Clostridiales. Cluster 2 contained two taxa, including [*Ruminococcus*] and *Collinsella* as well as *Dorea* and an unclassified member from the family *Lachnospiraceae*.

## DISCUSSION

The use of the Jersey breed of cattle in U.S. dairy systems has increased since the early 1990s, almost tripling to over 330,000 animals. This expansion in the breed's number was largely driven by their smaller size, reduced feed intake, and higher production of milk components (8), as well as individual perceptions, largely resulting from their small stature, that the breed is endearing, more docile, and thus easier to manage. While breed-specific comparisons in rumen microbial composition, fermentation efficiency, and biochemistry have been described between adult Jersey and Holstein cattle (16, 26), Jersey cows are found to harbor different taxa in their rumen microbiome, which intriguingly lends toward a differential production of VFAs and methane through metabolic pathways centered on reductant disposal via H_2_ producing pathways. The excess H_2_ is consumed by methanogens and reductive acetogens producing methane and acetate, respectively (16). However, the microbial makeup of Jersey calves remains unexplored, and we posit that the establishment of a microbial fermentation network distinct from Holsteins occurs in the neonatal period and influences long-term breed traits, including lipogenesis. To our knowledge, this study represents the first longitudinal characterization of the fecal microbiome in Jersey calves, providing insight into the temporal structure, stability, and potential functional implications of early microbial succession in this breed.

In both alpha and beta diversity metrics, age was the dominant force structuring the Jersey calf microbiome. Richness and Shannon diversity increased steadily with age beginning at 3 weeks of age, coinciding with a greater intake of solid feeds, ultimately converging toward dam-associated diversity levels by 12 weeks. Beta diversity analyses too demonstrated clear temporal structuring as calf fecal communities were initially very divergent from dams immediately after birth, forming a cluster from birth through 3 weeks of age and converging steadily with dams by the end of the study at 12 weeks of age. These patterns of convergence toward dams are not unique to Jersey calves in this study, as previous research from our group has consistently demonstrated this trend in Holstein calves (3, 4). The early divergence likely reflects the combined effects of milk-centered nutrition, variable environmental exposure, and seeding of oxygen-tolerant pioneer microbes. Whereas later convergence coincides with increased solid feed intake, noted at 3 weeks of age, rumen functional maturation, beginning with the introduction of hay and progressing rapidly post-weaning, and reduced inter-individual variability as diets and environments standardize. These pattern mirrors prior observations of microbial ecology in Holstein calves and beef breeds, where early rapid expansion of taxa is followed by stabilization as animals are weaned and the rumen matures (3, 4, 27–29).

Consistent with Furman et al., we identified a highly conserved core microbiome comprising 34 taxa, accounting for over 90% of total relative abundance and explaining more community variance across timepoints than non-core taxa. Core taxa clustered into six temporal phases that mirrored beta diversity and correlation patterns, suggesting that Jersey calf microbiome maturation proceeds through reproducible stage-specific states. However, because these transitions coincide closely with management changes in this cohort, it is difficult to disentangle the contribution of physiologic, environmental, and feed-related factors. Similar core-driven successional patterns have been reported in Holstein calves, supporting the idea that early-life microbial development in ruminants proceeds along defined paths largely constrained by host physiology, diet, and farm practices (2).

The earliest timepoint (24–48 h) was characterized by dominance of facultative anaerobes, particularly *Escherichia* and *Streptococcus*, together accounting for nearly half of the community abundance. This pattern is consistent with classic models of gut colonization and previous work in calves from our group and several others, wherein oxygen-tolerant taxa seed from the maternal skin, vaginal, and fecal microbiomes, forming niches that utilize oxygen to generate the anaerobic conditions required for other essential taxa ([1, 3, 4, 30–32]). The presence of *Blautia*, *Collinsella,* and *Dorea* at 14.6%, 6.9%, 2.8% relative abundance, respectively, in this early stage is notable as these genera are strictly anaerobic carbohydrate fermenters and previously are described at <1% relative abundance in the immediate post-natal stage (3–5, 7, 32). Their presence as the third, fourth, and sixth most prominent community members under non-ideal conditions, wherein oxygen tension is likely still elevated and primary fermenters have not yet established, indicates *Blautia, Collinsella,* and *Dorea* may be genetically linked to the Jersey breed and are heavily seeded in early life (31). The possibility of a genetic link between cow breed and microbial taxa is not unsubstantiated, as both *Blautia* and *Coriobacteriaceae*, of which *Collinsella* is a member, have been demonstrated as heritable taxa in beef cows (14). Throughout week 1, the fecal microbiome underwent a pronounced reorganization, marked by enrichment of milk- and host-glycan-utilizing taxa, *Lactobacillus*, *Enterococcus*, *Bacteroides*, at 13%–14% relative abundance each, alongside early butyrate producers *Faecalibacterium*, *Butyricoccus*, *Eubacterium*, and *Subdoligranulum,* representing 14.2% relative abundance collectively. Similar transient enrichments of these taxa have been observed in Holstein calves fed colostrum or milk-based diets and are thought to reflect utilization of lactose, milk oligosaccharides, and host-derived glycans (4, 5, 28). Interestingly, this stage also coincided with a sharp decline in *Collinsella* and *Blautia*, further suggesting that their immediate dominance may be seeded by the dam as calves were left with dams for the first 12 h of life, allowing for consistent transfer of maternal microbes through cow-calf contact. However, it remains to be determined if it is through skin contact via suckling, grooming behavior, the presence of maternal feces, or via birth through the vaginal microbiome and remaining fluids that the potential maternal seeding occurs. A reversion was observed at week 2 of age, as acetate-producing taxa, namely *Blautia*, *Collinsella*, and *Dorea*, reveal the spasmodic nature of microbial ecosystem formation, wherein environmental or host perturbations can have large effects on the evolving host-microbe and inter-microbial relationships as organisms compete for a niche. Other researchers comparing the microbiomes of Jersey and Holstein calves throughout the first 21 days of life demonstrated mixed results. Slanzon et al., found elevations in the abundance of *Lactobacillus* and *Streptococcus,* while Frazier et al., observed no differences in microbial ecology. However, direct comparisons with our work are difficult, as they undertook one to two samplings between 3 and 21 days, extracted DNA using different commercial kits, and amplified the V3–V4, and solely V4 regions of the 16S rRNA compared with our V1–V2 region (11, 12).

Weeks 3–7 were characterized by dominance of acetate-associated genera (*Blautia*, *Dorea*, *Collinsella*), with many belonging to the same network module and forming strong associations in correlation analyses. Similar temporal enrichment of *Blautia, Faecalibacterium, Collinsella,* and *Dorea* between 2 and 7 weeks of age has been reported in preweaning Holstein calves, with subsequent increases in unclassified Ruminococcaceae, Lachnospiraceae, and S24-7 as animals approach weaning (3–5, 32). The rise in these latter taxa is remarkably similar to data from our Jersey calves from week eight onward, wherein calves converged toward the maternal fecal microbiome, with enrichment of *Butyrivibrio*, *Turicibacter*, and unclassified members of S24-7, Ruminococcaceae, and Clostridiales, taxa associated with fiber degradation, secondary bile acid metabolism, and amino acid fermentation in mature ruminants (33, 34). Thus, the taxa involved in gut microbiome development and their trajectory are conserved across dairy breeds, reflecting the centrality of these taxa to calf gastrointestinal maturation. However, despite similarity in the presence of particular taxa, we observed differing abundance of acetate-associated taxa in Jersey calves. More specifically, *Blautia*, *Dorea*, and *Collinsella* represented at times nearly half of total community abundance, a prominence not described in any preweaning Holstein cohorts. Although direct comparisons with adult Jersey studies are limited by sample site differences (rumen vs. hindgut), research investigating differences in the Jersey and Holstein breeds demonstrated greater acetate and methane production in Jersey cows compared with Holsteins (16). These metabolite profiles were linked to enhanced hydrogen flux and greater potential for methanogenesis and reductive acetogenesis (16), matching the elevated abundance of acetate-associated taxa observed in our calves. Furthermore, despite its abundance, *Blautia* lacked strong network centrality in Jersey calves, suggesting its persistence may be driven more by breed-associated host genetics than by classical syntrophic dependencies with taxa such as *Faecalibacterium*, as shown previously in Holsteins (3, 4). That is not to say that *Blautia* does not play a distinguished role in the Jersey calf gut microbiome, as its inordinately high abundance demonstrates otherwise. Rather, if genetically driven, the genus may be an adaptation to the elevated H_2_ flux as seen in adult Jersey cattle, as within the genus several species of *Blautia* are reductive acetogens, thereby utilizing excess H_2_ and CO_2_ from the microbial community in turn providing additional acetate. Acetate, as the VFA best absorbed across the gut epithelium to the bloodstream for use peripherally, can then be used as a substrate for a growing calf (35 [16, 36]). Collectively, these findings suggest that the acetate- and H₂-biased metabolic phenotype described in adult Jerseys may originate during early-life microbial assembly.

Understanding the early-life origins of the gut fermentative milieu of different breeds is important, as it may offer periods at which researchers, veterinarians, and producers can intervene to direct energy from feedstuff to desired traits, whether they be reduced methane for sustainability or increased milk component production. The previous work demonstrating an elevated production of acetate in the Jersey cow rumen is of interest because, although SCFA concentrations were not measured, the high redundancy of acetate-producing taxa (*Blautia*, *Dorea*, *Collinsella*) suggests that there is elevated acetate production in Jersey calves during the preweaning period. In mammals, microbial acetate is absorbed across the gut epithelium and enters systemic circulation, where it serves as a primary substrate for peripheral lipid synthesis. In the mammary gland, acetate is the dominant precursor for *de novo* fatty acid synthesis, functioning through both substrate availability and signaling mechanisms (37; 38 [39]). Here, acetate activates GPCR43 and mTOR pathways, upregulating key lipogenic regulators including FAS, SREBP1, ACSS2, and PPARγ, thereby enhancing lipogenesis in bovine mammary epithelial cells (40 [41]). Beyond its metabolic role, acetate can modulate histone acetylation, linking microbial fermentation to epigenetic regulation of host metabolism and potentially increasing chromatin accessibility at loci involved in lipid synthesis (42). Importantly, the abundance of acetate-associated taxa in Jersey calves occurs during a critical developmental window for the mammary gland. Elevated planes of nutrition during the preweaning period have been shown to increase mammary parenchymal mass and alter subsequent milk yield (43–46). Thus, elevated systemic acetate availability during early life may impact metabolic and epigenetic signaling during mammary tissue development. While speculative, this raises the possibility that the acetogen-centric microbial community in Jersey calves imprints the long-term H₂-driven metabolic phenotype described in adult Jerseys (16), ultimately influencing milk fat synthesis of the Jersey breed. This begs the question: as Holstein milk fat percentages continue to rise through genetic selection, are microbial profiles indirectly selected for as well? Future comparative longitudinal studies integrating host-microbe genomics, metabolomics, and parallel breed cohorts will be essential to determine whether microbial ecology is causally linked to trait selection.

Alternatively, given Jersey calves’ smaller stature, they possess a higher surface area-to-mass ratio, thus losing a greater proportion of energy to heat transfer (47), and the dominance of acetate-associated taxa may act as a mechanism for thermoregulation. Acetate has been found to be the primary driver of lipogenesis in calf and lamb brown adipose tissue (BAT; (48) 35). BAT undergoes β-oxidation of fats, dissipating chemical energy as heat via uncoupling protein-1, and trials using mice have demonstrated that exogenous acetate feeding increases GPCR43, triggering increased presence of beige fat and thermogenesis (49). With differential responsiveness to lipogenic acetate signals established in cattle (48), Jersey calves may require excess acetate production from the microbiome, which in turn is directed into BAT and contributes to non-shivering thermogenesis necessary for the survival of the smaller calves (48).

While this study provides the first temporal profile of the maturation of the Jersey calf gut microbiome, it has some limitations. The smaller sample size (12 calves) and single-farm design may restrict generalizability across management systems or genetic lines. Tracking direct fermentation metrics, such as short-chain fatty acids and gas production, would provide clarity regarding Jersey microbial metabolism of the breed. Moreover, the analyses herein were based on 16S rRNA data; future work integrating shotgun genomics and targeted metabolomics would increase granularity with regard to prominent species and metabolic pathway abundance. Studies performing parallel breed comparisons *in vitro* and *in vivo* will be critical to disentangle microbial, dietary, and host differences between calf breeds. Our findings demonstrate that the Jersey calf hindgut microbiome undergoes a structured, age-dependent maturation, characterized by the early dominance of acetogenic taxa, a conserved core microbial succession, and the emergence of adult-associated taxa, with gradual convergence to an adult microbial ecosystem. The results herein lay the foundation for future investigations into how early-life microbial succession may contribute to lifelong health and productivity in Jersey cattle.

## Data Availability

The 16S rRNA raw sequence data have been submitted to the NCBI database under BioProject accession number PRJNA1435288.

## AFTER EPUB

*[This article was published on 9 October 2026 with an error in the title. The title was corrected in the current version, posted on 16 October 2026.]*

## SUPPLEMENTAL MATERIAL

Fig. S1 spectrum.01878-26-s0001.eps Stacked bar plots of phyla and genus (CalfvsDam).

Fig. S2 spectrum.01878-26-s0002.eps Heatmap of core taxa across time.

Supplemental legends spectrum.01878-26-s0003.docx Legends for Fig. S1 and S2, and Table S1.

Table S1 spectrum.01878-26-s0004.csv Relative abundance proportions of all taxa.

ASM does not own the copyrights to Supplemental Material that may be linked to, or accessed through, an article. The authors have granted ASM a non-exclusive, world-wide license to publish the Supplemental Material files. Please contact the corresponding author directly for reuse.
