# Supplementary material for: Core microbial populations drive longitudinal development of the hindgut bacterial microbiota in Jersey calves
Source: Microbiol Spectr. 2026 Oct 9;14(11):e01878-26. doi: 10.1128/spectrum.01878-26 (PMC13729157; doi:10.1128/spectrum.01878-26)
Supplement: Supplementary file 1 [file spectrum.01878-26-s0003.docx]

**Supplementary Figure 1.** Stacked bar plots of relative abundance of dominant bacterial phyla (A) and genera (B) in Jersey calves and dams. Individual results of the calves (n=12) and dams were grouped by age and category (whether the animal was a dam or calf) for analysis.

**Supplementary Figure 2.** Heatmap of core taxa (prevalence >80%, mean relative abundance >0.5%) based on rarefied counts. Samples were clustered using Euclidean distance and Ward’s D2, and cluster number determined by gap statistic. Color scale indicates relative abundance from low (blue) to high (red).

**Supplementary Table 1.** Values represent the mean and standard deviation of percentage relative abundance of each detected taxon within each sampling timepoint from calves grouped by age.
